# Daylight Saving Time is not Associated with an Increased Number of Trauma Activations

**DOI:** 10.5811/westjem.2019.5.42780

**Published:** 2019-07-02

**Authors:** David Chung-Sang Lee, Barbara A. Stahlman, Mark L. Sharrah

**Affiliations:** * WellSpan York Hospital, Department of Emergency Medicine, York, Pennsylvania

## INTRODUCTION

Prior studies have reported that during the period of Daylight Saving Time (DST) adjustment, there are a greater number of motor vehicle collisions.^1^–^3^ These studies noted that the increase in collisions occurs mainly on the following Monday after the time change. We hypothesize that changes in sleep patterns may be one of the reasons for this increase. Although the number of fatal accidents increases, it is unclear whether there is an increased need for hospital-based trauma services. We hypothesized that there may be an increased number of trauma activations during DST changes that may indicate a need for increased resources.

## METHODS

We performed a retrospective chart review at an academic, tertiary care hospital identified as a Level 1 trauma center. This hospital serves multiple counties and has an emergency department annual census of 83,000 patients. There are no other trauma centers in the primary county. Data, including dates and total number of trauma activations for those respective dates, were extracted from the hospital’s trauma registry. We compared the number of trauma activations occurring on the Mondays of DST change to those occurring on the Mondays one week before and one week after the DST changes, over a 20-year span. Mann-Whitney U tests were employed for all analyses.

## RESULTS

At the start of DST, the median number of trauma activations (N = 40) on the Mondays of DST (median = 2, range = 7) did not differ significantly (U = 190.5; p = 0.41) from the Mondays one week before (median = 2.5, range = 7), nor did it significantly differ (U = 184; p = 0.34) from the Mondays one week after (median = 2.5, range = 7). Likewise, at the end of DST, the median number of total trauma activations for the Mondays of DST (median = 2, range = 7) did not differ significantly (U = 167; p = 0.19) from the Mondays one week before (median = 3, range = 7), nor did it significantly differ (U = 161.5; p = 0.15) from the Mondays one week after (median = 2, range = 7).

## DISCUSSION

We did not identify an increase in the number of trauma activations associated with DST changes during the study period. Our hospital is located in the Mid-Atlantic area of the United States and our data might differ from other sites by experiencing greater lighting changes during DST. Furthermore, with a relatively low sample size of trauma activations per day, there may not be enough variability to detect changes associated with DST. Although this site provides care to multiple counties with the nearest Level 1 trauma center over 20 miles away, we did not find a significant increase in activations. Currently, there are no indications for increasing trauma services during DST changes at our single site.[Table t1-wjem-20-585]

## Figures and Tables

**Table 1 t1-wjem-20-585:** Trauma activations on Mondays.

	Median Number of Activations	Range	P
Mondays 1 week before start of DST	2.5	7	0.41
Mondays of start of DST	2.0	7	
Mondays 1 week after start of DST	2.5	7	0.34
Mondays 1 week before end of DST	3.0	7	0.19
Mondays at end of DST	2.0	7	
Mondays 1 week after end of DST	2.0	7	0.15
